# 3’*Igh* enhancers hs3b/hs4 are dispensable for *Myc* deregulation in mouse plasmacytomas with T(12;15) translocations

**DOI:** 10.18632/oncotarget.26160

**Published:** 2018-10-02

**Authors:** Alexander L. Kovalchuk, Tomomi Sakai, Chen-Feng Qi, Wendy Du Bois, Wesley A. Dunnick, Michel Cogné, Herbert C. Morse

**Affiliations:** ^1^ Virology and Cellular Immunology Section, Laboratory of Immunogenetics, National Institute of Allergy and Infectious Diseases, National Institutes of Health, Rockville, MD, USA; ^2^ Animal Model and Genotyping Core Facility, Laboratory of Cancer Biology and Genetics, NCI, National Institute of Health, Bethesda, MD, USA; ^3^ Department of Microbiology and Immunology, University of Michigan, Ann Arbor, MI, USA; ^4^ Laboratory of Immunology, CNRS UMR 7276, Université de Limoges, Limoges, France

**Keywords:** immunoglobulin, Myc, translocation, PCT

## Abstract

*Myc*-deregulating T(12;15) chromosomal translocations are the hallmark cytogenetic abnormalities of murine plasmacytomas (PCTs). In most PCTs, the immunoglobulin heavy chain (*Igh*) locus is broken between the *Eμ* enhancer and the 3’ regulatory region (*3’RR*), making the latter the major candidate for orchestrating *Myc* deregulation. To elucidate the role of the *Igh3’RR* in tumorigenesis, we induced PCTs in *Bcl-xL-*transgenic mice deficient for the major *Igh3’RR* enhancer elements, *hs3b* and *hs4* (hs3b-4^-/-^). Contrary to previous observations using a mouse lymphoma model, which showed no tumors with peripheral B-cell phenotype in hs3b-4^-/-^ mice, these animals developed T(12;15)-positive PCTs, although with a lower incidence than hs3b-4^+/+^ (wild-type, WT) controls. In heterozygous hs3b-4^+/-^ mice there was no allelic bias in targeting *Igh* for T(12;15). Molecular analyses of *Igh/Myc* junctions revealed dominance of *Sμ* region breakpoints versus the prevalence of Sγ or Sα in WT controls. *Myc* expression and Ig secretion in hs3b-4^-/-^ PCTs did not differ from WT controls. We also evaluated the effect of a complete *Igh3’RR* deletion on *Myc* expression in the context of an established *Igh*/*Myc* translocation in ARS*/Igh*11-transgenic PCT cell lines. Cre-mediated deletion of the *Igh3’RR* resulted in gradual reduction of *Myc* expression, loss of proliferative activity and increased cell death, confirming the necessity of the *Igh3’RR* for *Myc* deregulation by T(12;15).

## INTRODUCTION

The two major regulatory elements in the *Igh* locus are the *Eμ* intronic enhancer that promotes V(D)J recombination in developing B-cells, and an enhancer cluster located downstream of all constant gene segments, termed the 3’ regulatory region (*3’RR*), and recently classified as a superenhancer, which has a crucial functional role [1, 2]. The *Igh3’RR* contains four individual enhancer units coinciding with DNAse I hypersensitive sites hs3a, hs1,2, hs3b and hs4 followed by a cluster of CTCF sites, the CTCF superanchor [3, 4, 5]. hs3a and 3b enhancers are part of the *Igh3’RR* quasi-palindrome or proximal enhancer module with correct functioning dependent on the presence of inverted repeats [1]. The individual enhancers differentially bind a plethora of transcription factors and are maturation stage-specifically activated during B-cell development (Reviewed in [6]). The whole *Igh3’RR* region is essential for somatic hypermutation (SHM) and class switch recombination (CSR) as well as high level Ig expression in plasma cells (Reviewed in [7, 8]). Any alteration in the architecture of the *Igh* superenhancer does affect its functions (reviewed in [6]).

CSR and SHM are mediated by AID [9]. Misdirection of AID to various loci may result in chromosomal translocations [10] leading to immunoglobulin superenhancers invading and deregulating expression of the other gene’s domains. Only those translocations that target oncogenes will lead to clonal growth of malignant tumors with mature B-cell or plasma cell phenotypes. In humans, the list of partners for *Igh* translocations in tumors is rather extensive, but is primarily limited in mice to the *Myc* oncogene with the translocated chromosome designated T(12;15).

Terminally-differentiated mouse B-cell tumors that carry *Ig/Myc* translocations – plasmacytomas (PCT) - can be induced by intraperitoneal injections of BALB/c mice with pristane [11]. In the presence of an *Eμ-Bcl-xL* transgene, pristane-induced PCT arise in an accelerated manner and are not dependent on the BALB/c genetic background [12]. The anti-apoptotic action of the *Bcl-xL* transgene may extend the lifespan of tumor precursors, allowing them to acquire additional secondary oncogenic changes. In 80-85% of these tumors, *Myc* is juxtaposed to a switch region of the *Igh* locus, thus removing the *Eμ* enhancer from the *Myc*-containing chromosome [T(12;15)]. The remaining 10-15% have *Igκ or Igλ* light chain to *Pvt-1* translocations, T(15;16) or T(6;15), and less than 5% of the tumors contain either insertions of the *Eμ* enhancer or other insertions/rearrangements in the 5’ *Myc* promoter region [13]. In the IL-6 transgenic spontaneous PCT model, we described otherwise rare cases of T(12;15) junctions that have breakpoints clustering around the J_H_4 segment, thus retaining the *Eμ* enhancer on the *Myc*-containing chromosome [14, 15].

There are numerous mouse models that result in the development of B-cell or plasma cell tumors with the T(12;15) translocation (Reviewed in [16]). Some of them employ mutant mice with defects in DNA repair pathways that generally develop pro-B lymphomas where *Myc* expression is under the control of the *Eμ* enhancer [17, 18]. By combining several mutations, it was also possible to obtain lymphomas with peripheral B-cell phenotype in which *Myc* was joined with an *Igh* switch region sequence and the *Eμ* enhancer was deleted, leaving *Myc* under the control of the *Igh3’RR* alone [19].

The crucial role of *Igh*3*’RR* in B-cell lymphoma development and *Myc* deregulation was studied extensively employing various types of transgenic or knock-in models (Reviewed in [20]). An alternative approach involves induction of T(12;15) translocation-positive tumors in mice carrying various deletions of the *Igh3’RR* region. It was shown that mice prone to lymphoma development (due to a combined p53 and DNA Ligase 4 defect) and simultaneously lacking hs3b-4 enhancers overwhelmingly succumbed to pro-B cell lymphomas [21]. In such pro-B cell lymphomas, *Myc* expression was likely deregulated by the *Eμ* enhancer since the *Igh*3’*RR* is poorly active at this stage. In the same study, a different mouse model of conditional inactivation of XRCC4 in transitional B-cells of p53-deficient mice was also utilized. These mice are ordinarily predisposed to peripheral B-cell lymphomas and those arose only on an *Igh*3’*RR* WT background. The characteristic T(12;15) translocation-positive cells were detectable but were not selected for further tumor progression, due to the lack of *Myc* deregulation in the case of a truncated *Igh3’RR*.

The function of *Igh*3’*RR* individual enhancer units changes through different stages of B-cell differentiation with hs1,2 enhancers sequentially gaining higher activity during the GC and plasma cell stages [22]. Also, the model described in the previous study utilized a NHEJ- and p53-deficient background that strongly favors peripheral lymphomas originating from transitional B-cells. We thus decided to re-assess the effect of the same hs3b-4 deletion on the development of tumors with terminally differentiated B-cell phenotypes using a NHEJ- and p53-proficient mouse PCT model. We injected hs3b-4-deficient (hs3b-4^-/-^) mice carrying the *Eμ-Bcl-xL* transgene with pristane. If hs3a/1,2 were insufficient to deregulate *Myc* expression, one might expect them to develop PCTs carrying T(6;15) or T(15;16) *Ig* light chain/*Myc* translocations or *Eμ* insertions into *Myc* (perhaps at a lower rate). However, hs3b-4^-/-^ mice developed PCTs with the vast majority of the *Igh/Myc* breakpoints located downstream of *Eμ.* The pattern of breakpoint distribution along the *Igh* locus and tumor incidence differed from normal controls, with differences likely relevant to the accessibility of the *Igh* locus to switch recombination. We conclude that hs3b-4 enhancer is crucial for synapsis with various parts of the *Igh* locus at the initiation of translocations in mature B-cells and for subsequent *Myc* deregulation, but then becomes dispensable once a translocation-positive precursor cell differentiates to the plasma cell stage.

If such a “hypomorphic” *Igh3’RR* with a partial deletion can support *Myc* deregulation in plasma cell tumors, is it then possible that the complete hs1,2,3a,3b,4 combination of *Igh3’RR* enhancers is no longer essential in established PCTs? In a second model using established PCT cell lines, we show that the *Igh3’RR* still crucially controls the tumor cell phenotype. Indeed, when the whole hs1,2,3a,3b,4-containing region was deleted from a *Myc*-translocated chromosome using Cre recombinase, deregulated *Myc* expression could no longer be sustained.

## RESULTS

### hs3b-4^-/-^ mice develop PCT with reduced incidence

**Figure 1:**
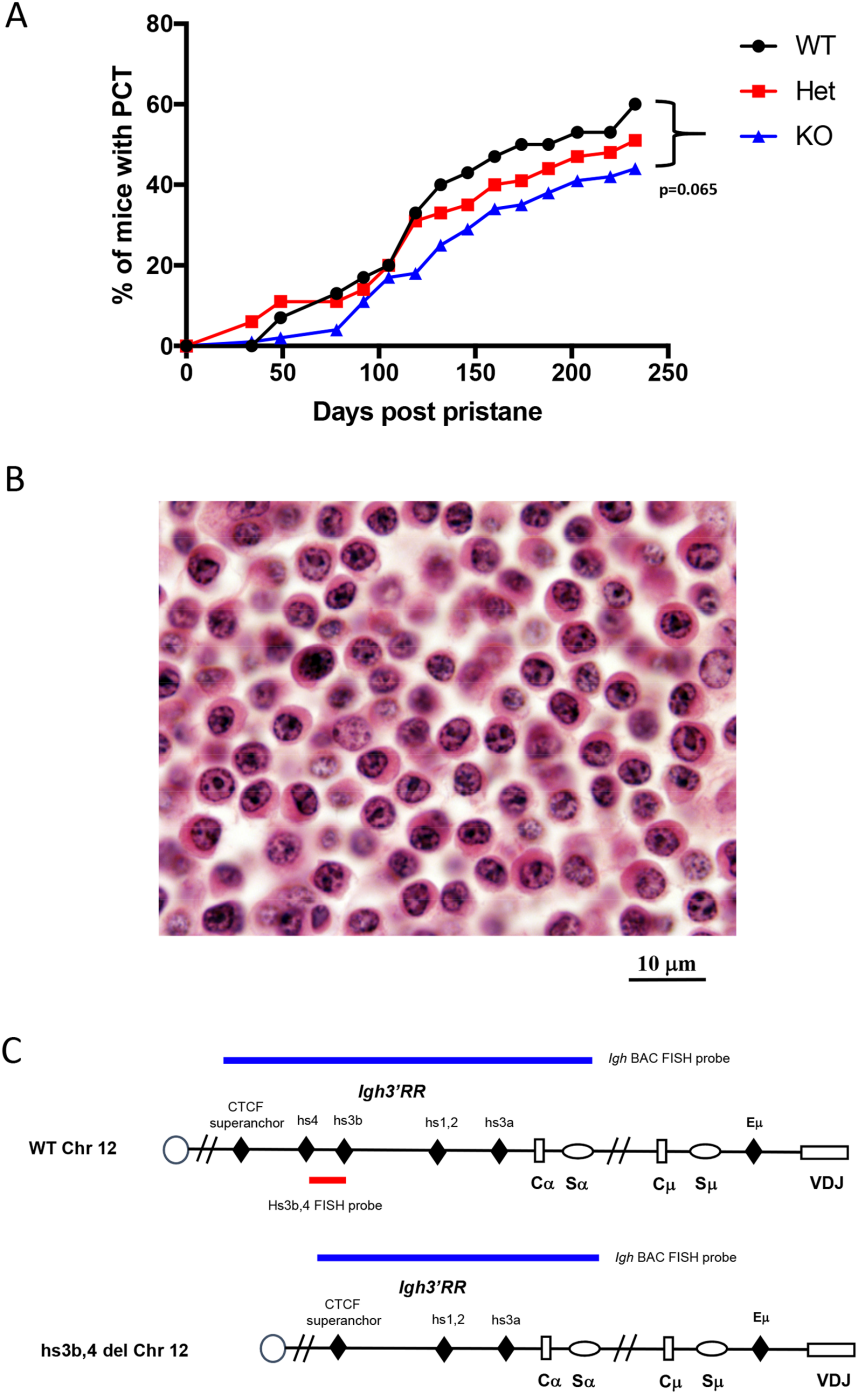
Deletion of hs3b-4 only moderately reduces PCT tumor incidence **(A)** Incidence of PCT in hs3b-4^+/+^, hs3b-4^+/-^ and hs3b-4^-/-^ mice. The graph shows a lower tumor incidence in the hs3b-4^-/-^ group and a haploinsufficiency phenotype in the hs3b-4^+/-^ group. **(B)** Photomicrograph of oil granuloma showing advanced growth of a hs3b-4^-/-^ PCT. The tumor has plasmablastic/plasmacytic appearance with abundant eosinophilic cytoplasm and a PCT-typical clock-face nuclear pattern (H&E, 100x oil). **(C)** Schematics of the WT and hs3b-4-deficient *Igh* locus. Colored horizontal bars depict location of FISH probes used to detect *Igh* translocations. 3’*Igh* BAC probe detects both WT and deleted alleles. hs3b-4 PCR-amplified probe detects only WT allele.

Homozygous hs3b-4^-/-^ mice were previously found to have defects in isotype switching [23] and, on p53- and NHEJ-deficient background, they developed pro-B rather than peripheral B-cell lymphomas with *Igh/Myc* translocations [21]. Here, we investigated the effect of this mutation on the occurrence and the pattern of chromosomal translocations in pristane-induced PCT, a model in which lymphomagenesis is known to focus on terminally differentiated B-cells. Three groups consisting of 30 hs3b-4^+/+^, 66 hs3b-4^+/-^ and 124 hs3b-4^-/-^ mice, each also carrying an anti-apoptotic *Bcl-xL* TG, were injected i.p. once with 0.4 ml of pristane. Appearance of atypical plasma cells in peritoneal washouts was interpreted as an indicator of tumor development. All three groups of mice rapidly developed PCT with mean latencies of 118, 128 and 129 days, respectively (Figure 1A). We observed a haploinsufficient phenotype as the tumor incidence was lower in the hs3b-4^+/-^ (51%) than in the hs3b-4^+/+^ (60%) cohort. Even a lower proportion of hs3b-4^-/-^ mice (44%) had developed PCTs at day 240 [p=0.065].

Histologic analyses of sections prepared from oil granuloma tissues revealed characteristic atypical plasma cells with high proportion of plasmablasts in the tumors and no apparent differences in cytomorphology between experimental groups (Figure 1B).

In addition, there were no differences from WT in the ability of hs3b-4^-/-^ or hs3b-4^+/-^ tumors to grow as transplants in pristane-primed nude mice or in their adaptability to grow *in vitro* (16 hs3b-4^-/-^ and 13 hs3b-4^+/-^ primary (G0) or first-generation transplant (G1) tumors were established as cell lines (Supplementary Table 1)).

### PCTs in hs3b-4^-/-^ mice carry reciprocal *Igh/Myc* translocations

**Figure 2:**
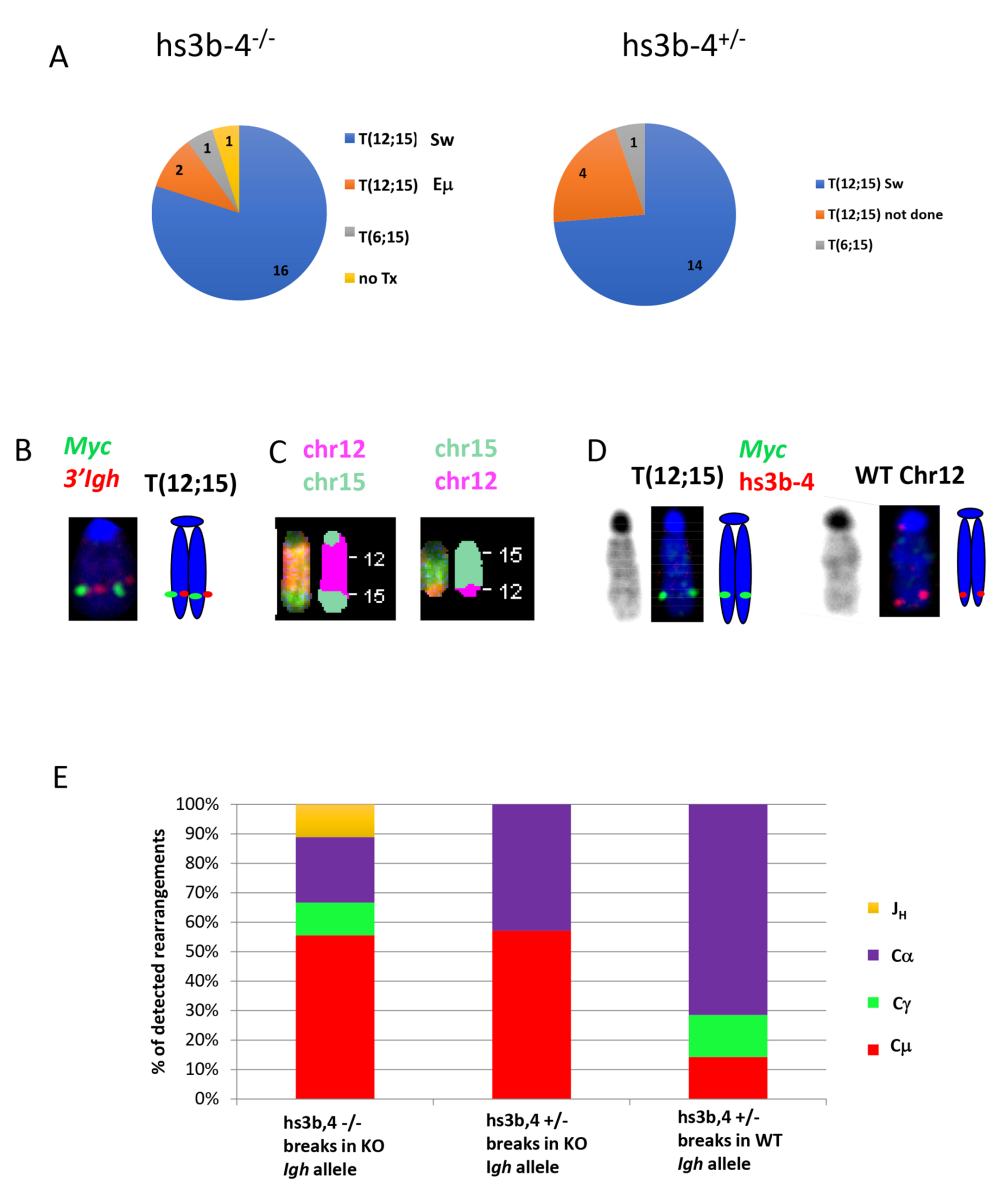
In mouse PCTs, deletion of hs3b-4 does not affect the pattern of chromosomal translocations but significantly increases targeting of Sμ for T(12;15) **(A)** Frequency of PCT tumors on hs3b-4^-/-^ (left) or hs3b-4^+/-^ (right) background with T(12;15) breakpoints within *Igh* switch regions; with T(12;15) breakpoints upstream of *Eμ*; with variant translocations T(6;15) or without *Ig*/Myc translocations. Chart shows predominance of tumors with T(12;15) with breakpoints in switch regions in both groups. **(B)** Cytogenetic findings in regards to T(12;15) translocations in the hs3b-4^-/-^ group. Left – FISH with *Igh* probe (red) and *Myc* probe (green). Signals co-localize at the telomeric portion of Chr. 12. Right - graphic representation. **(C)** SKY images of Chr 12;15 and 15;12. **(D)** Targeting hs3b-4 KO allele by T(12;15) in hs3b-4^+/-^ tumors. FISH using simultaneous staining with *Myc* (green) and hs3b-4 probes (red). The hs3b-4 probe hybridizes only to the intact WT Chr 12 (right). The *Myc* probe hybridizes to normal Chr 15, Chr T15;12 (both not shown here but can be found on Supplementary Figure 2) and to longer translocated Chr T(12;15) that has deletion in the *Igh3’RR* (left). Right - graphic representation. Left – DAPI staining. **(E)** Distribution of *Igh*/Myc breakpoints within *J*_*H*_, *Cμ*, *Cγ* (combined *Cγ2a* and *b*) and *Cα*. Shows prevalence of *Cμ* breakpoints in the hs3b-4^-/-^ group and *Cα* in the WT group.

The *Igh3’RR* enhancer-competent *Bcl-xL*-transgenic mice develop PCTs that deregulate *Myc* by virtue of *Igh/Myc* T(12;15) chromosomal translocations with corresponding breakpoints located predominantly in the Sα region of *Igh* and in close proximity to the P1 promoter of *Myc* [30]. We investigated whether PCTs from hs3b-4^-/-^ mice utilized this conventional pathway or developed variant *Igk* or *Igλ* translocations, (T(6;15) and T(16;15) respectively), to the *Pvt-1* locus. We first applied FISH and SKY techniques to determine whether the tumors carried any *Ig/Myc* translocations. By FISH, 21 out of 23 PCTs from the hs3b-4^-/-^ group and 20 of 21 PCTs in the hs3b-4^+/-^ group carried a reciprocal T(12;15) (Figure 2A and Supplementary Table 1). A single tumor in the hs3b-4^-/-^ group lacked detectable translocations involving either *Ig*- or *Myc*-containing chromosomes. This PCT harbored a rare, for this type of tumor, MuLV integration in *Myc* intron 1, as detected by transcript fusion analysis using RNA-seq (Supplementary Table 2). Both groups had a single case with a T(6;15). Using SKY on selected cell lines, we confirmed the presence of reciprocal T(12;15) translocations that were initially identified by FISH (Figure 2B and 2C, Supplementary Figures 1 and 3). All analyzed tumors had simple diploid or near tetraploid karyotypes with very few additional non-recurrent chromosomal aberrations.

### Translocations in hs3b-4^+/-^ mice have no allelic bias

It was shown earlier in a lymphoma model that only the WT allele was involved in T(12;15) [21]. Using previously described techniques, we asked if there was any bias in translocation targeting of *Igh* alleles in hs3b-4^+/-^ mice. We analyzed 15 hs3b-4^+/-^ tumors and found no significant differences in allele utilization. In seven cases, *Myc* was translocated into the WT *Igh* chromosome and in nine cases into the KO allele (Figure 1D, Supplementary Figure 2 and Table 1). Interestingly, in three of the latter cases, the untranslocated WT Chr. 12 was lost and the hs3b-4 KO allele-containing T(12;15) was duplicated (Supplementary Figure 3). The fact that these tumors still possessed a normal Chr. 15 and a single copy of T(15;12) precluded the possibility of two independent translocation events involving both *Igh* alleles.

### The distribution of translocation breakpoints in the hs3b-4-deficient *Igh* allele is indicative of their canonical nature but with overutilization of breaks in the *Sμ* region

To precisely map *Igh/Myc* translocation breakpoints, we utilized a long PCR-based screening method [31]. This method allows for identification of specific *Igh* C regions adjacent to the translocation breakpoint. In addition, we screened for junctions containing the *Eμ* enhancer in close proximity to *Myc* coding regions. The latter type of rearrangement is permissive for *Myc* deregulation in the absence of *Igh3’RR* enhancers and has been described for rare classical and IL-6 transgenic PCTs [32].

All analyzed tumors appeared to have a single dominant clone as only a pair of T(12;15) and T(15;12) products was amplified from each sample in one round of PCR amplification. We did not attempt to explore the presence of other minor clones.

The *Myc* breakpoints spanned a narrow region just upstream of the P1 promoter, which is typical for *Bcl-xL*- and *Bcl2*-transgenic PCTs [30, 33] (Supplementary Table 2). Breakpoints in *Igh* in hs3b-4^+/-^ tumors with translocations into the WT allele followed the pattern described earlier for WT tumors [30]. Sα was targeted in more than 70% of cases, with breakpoints in Sγ and Sμ being in the minority (Figure 2E, Supplementary Figure 4).

Breakpoints the KO allele in both hs3b-4^-/-^ and hs3b-4^+/-^ tumors showed a pattern drastically different from WT (breakpoints in WT *Bcl-xl*-transgenic PCTs were described in [30]). The majority of breakpoints (56%) were in the *Sμ* region. *Sα* was also targeted, but much less frequently (22%). Two exceptional cases in the hs3b-4^-/-^ group (79923, 80585) had breakpoints in *Jh* segments and retained the *Eμ* enhancer on T(12;15) in close proximity to *Myc* promoters, possibly abolishing a requirement for a fully active *Igh3’RR* enhancer for deregulation of the translocated *Myc* (Supplementary Table 2). No *Igh/Myc* junctions were detected by PCR with any primer combinations in 4 out of 21 hs3b-4^-/-^ and in 2 out of 13 hs3b-4^+/-^ otherwise T(12;15)-positive samples. This is consistent with previous observations regarding the efficiency of our PCR-based translocation detection method [31]. Regardless of the exact location of the translocations that are not detectable by the PCR primers we used, it is unlikely that they would alter the striking differences between WT and hs3b-4-deficient *Igh* alleles.

### Analyses of VDJ rearrangements and switch region junctions

In the process of obtaining *Igh/Myc* breakpoint sequences specific for the reciprocal Chr (15;12), we extended PCR-defined boundaries to also include VDJ- and DJ-junctions. These, together with switch region junctions, can be used as additional markers for identification of individual *Igh*-bearing chromosomes and for matching FISH and PCR results. We sequenced VDJ rearrangements for 14 hs3b-4^-/-^ and 16 hs3b-4^-/-^ tumors (Supplementary Table 3). There was no differential utilization of particular V-region families. 36% of hs3b-4^-/-^ tumors appeared to lose Ig expression as a non-productive, out-of-frame VDJ gene segment was located on the non-translocated Chr 12 and the other *Igh* allele was interrupted by T(12;15). This is frequent in PCTs arising in *Bcl-xL*-transgenic mice where enforced expression of the anti-apoptotic protein, Bcl-xL, rescues PCT precursor cells with abrogated BCR signaling.

In 9 cases of hs3b-4^-/-^ PCTs, we were able to identify a C region that partnered with a rearranged VDJ segment. Isotype switching did occur on un-translocated *Igh* alleles and its pattern mostly matched the distribution of T(12;15) breakpoints in hs3b-4^-/-^ PCTs. Non-switched alleles (VDJ-Cμ) were identified in 44% of the cases, with Cα and Cγ2b also being frequently utilized (56%). In hs3b-4^+/-^ tumors, out of three analyzed cases where the non-translocated Chr 12 was deprived of hs3b-4, two remained unswitched. We also analyzed switch junctions from four hs3b-4^-/-^ tumors and found them to be typical (Supplementary Table 4). This is in agreement with observations previously made in non-malignant B-cells with a 3’RR defect [1].

### hs3b-4^-/-^ PCTs overexpress *Myc* and exhibit the P2 to P1 promoter shift

**Figure 3:**
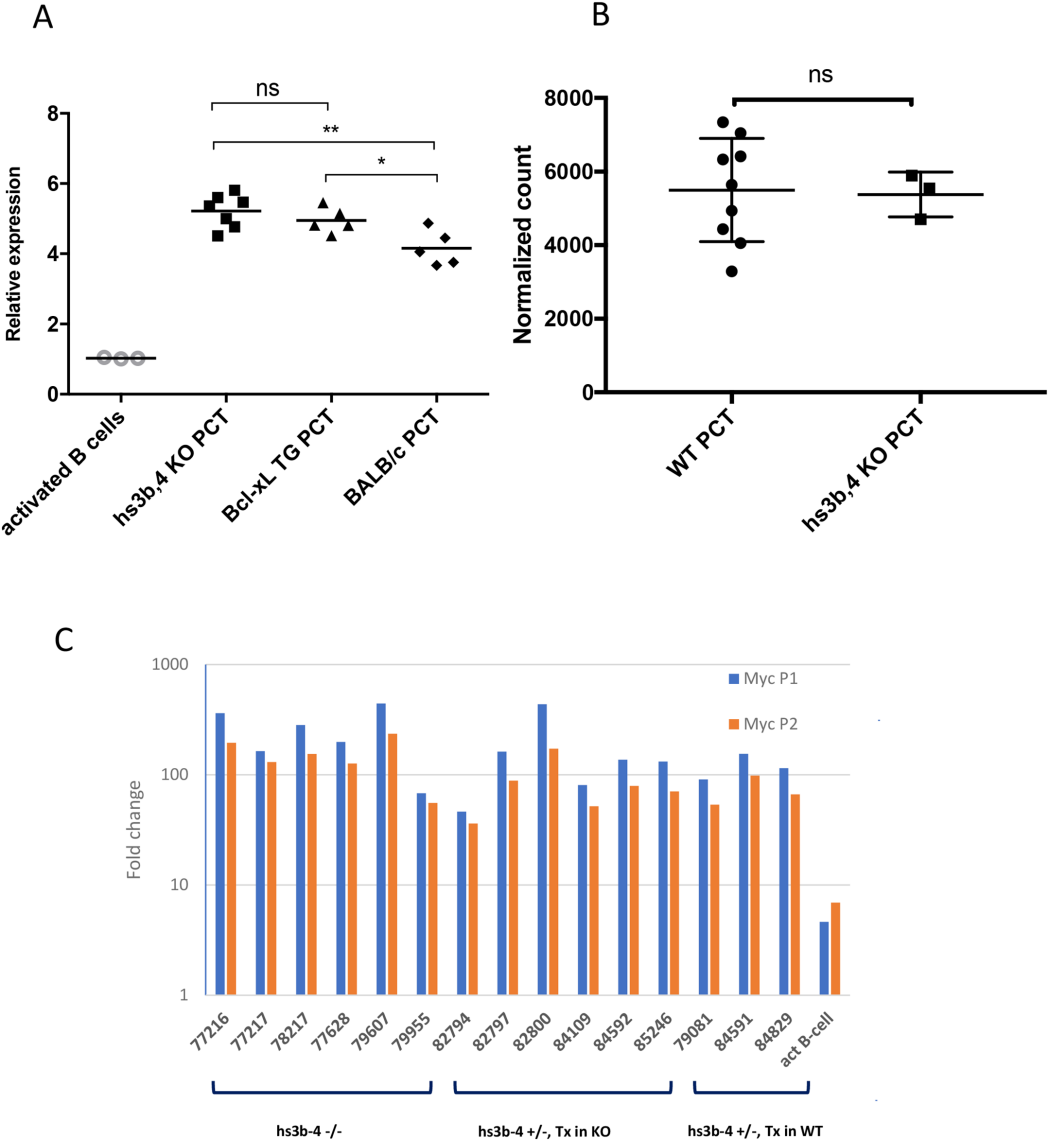
hs3-4b deficiency does not affect *Myc* expression in PCTs **(A)** qPCR analysis of *Myc* expression normalized to β-actin. hs3b-4^-/-^, WT *Bcl-xL*-transgenic and classical Balb/c PCT cell lines were compared to non-transgenic IL-4 and LPS-activated B-cells. Transcripts spanning coding exons of *Myc* were detected using Exon 2F and Exon 3R primers. Values in activated WT B-cells were set to 1. Horizontal line represents mean expression level within a group. **(B)** Mean normalized expression of *Myc* in WT and hs3b-4^-/-^ samples as detected by RNA-seq. **(C)** qPCR analysis of *Myc* P1/P2 promoter usage in mouse PCTs. Levels of transcripts initiating at the P1 promoter were compared to the total message detected downstream of the P2 promoter. Fold change was calculated against the corresponding levels in resting B-cells. hs3b-4^-/-^ and hs3b-4^+/-^ cell lines with both KO and WT translocation-targeted *Igh* alleles were included in the analysis. Activated B-cells from WT control were also included. ^*^p<0.05, ^**^p<0.01, ns – not significant

We analyzed relative levels of *Myc* expression in hs3b-4^-/-^ PCT compared to those of *Bcl-xL*-transgenic PCTs and non-transgenic PCTs. *Myc* expression fold change was calculated relative to that of IL-4 and LPS-activated mixed genetic background WT CD43^-^ splenic B-cells. As expected, all PCT cell lines exhibited drastically elevated *Myc* mRNA compared to IL-4 and LPS-activated splenic B-cells. There were no significant differences in *Myc* mRNA levels between hs3b-4^-/-^ and WT *Bcl-xL*-transgenic PCTs (Figure 3A). However, *Bcl-xL*-transgenic PCTs had significantly higher levels of *Myc* expression (p=0.04) than non-transgenic Balb/c PCTs. It is plausible to suggest that anti-apoptotic action of *Bcl-xL* allows transgenic PCTs to sustain the highest *Myc* mRNA levels. These findings were reconfirmed using RNA-seq (Figure 3B).

Earlier, it was proposed that the hs4 enhancer governs P2 to P1 *Myc* promoter shift in lymphoma cells [34]. We decided to determine the effect of hs3b-4 deletion on PCT-specific *Myc* promoter shift [35]. The fact that all hs3b-4^-/-^ tumors had breakpoints in *Myc* just upstream of the P1 promoter allowed us to analyze *Myc* promoter utilization.

*Myc* promoter usage was estimated by comparing transcripts originating from P1 (detected using mmu-MYC P1F and mmu-MYC Exon2R primers) and the sum of transcripts originating at both P1 and P2 (detected using mmu-MYC P2F and mmu-MYC Exon2R primers). As a baseline, we used *Myc* transcription level in normal B-cells where it is expressed predominantly from the P2 promoter. Specificity of PCR products was assessed by agarose gel electrophoresis.

Again, there were no significant differences among the groups. All analyzed PCTs with breakpoints upstream of *Myc* promoters exhibited the P2 to P1 promoter shift. As shown on Figure 3C, message originating from P1 prevailed over that from P2 and the relative ratio of P1/P2 message abundance was greater than one.

This finding was reconfirmed using RNA-seq of two hs3b-4^-/-^ PCTs: 77210 and 77628. RNA-seq-detected levels of PCT-specific genes including *Myc* and plasma cell markers (*Prdm1*, *Sdc1* and others), did not differ significantly from WT controls (data not shown). Analysis of RNA-seq reads shows robust initiation of *Myc* transcription from the P1 promoter (Supplementary Figure 5). Also, a minority of reads spanned an *Igh*/*Myc* translocation breakpoint and contained junction sequences identical to the ones detected by PCR on genomic DNA. These chimeric *Igh/Myc* transcripts are typical for mouse PCTs and Burkitt lymphomas [36].

### Secretion of paraproteins is not significantly affected in hs3b-4^-/-^ PCTs

Electrophoretic studies were performed with sera obtained from mice with primary (G0) or transplanted (G1) tumors. 48% hs3b-4^-/-^ (20 out of 42) and 63% hs3b-4^+/-^ (5 out of 8) primary PCTs showed single or multiple clonal bands in the gamma mobility assay (Supplementary Table 5).

Fifteen hs3b-4^-/-^ and two hs3b-4^+/-^ samples with the most prominent M components were selected for further immunochemical analysis using antibodies specific to all immunoglobulin heavy chain classes and subclasses except IgE. Immuno-isotyping showed that all but 13 samples from both groups contained a single dominant paraprotein. IgA and IgG1 were prominent once each, IgM twice, IgG2a three times and IgG2b seven times. Paraprotein titers were high and, in many cases, exceeded 1.28 × 10^−5^. These findings indicate that hs3b-4^-/-^ PCTs can secrete large amounts of paraproteins. This observation is reminiscent of the fact that a defect in Ig secretion by non-malignant plasma cells was mostly observed in mice with a complete *Igh*3’RR deletion [37] but not with the partial deletion eliminating hs3b and hs4 only [23].

### Cre-mediated deletion of hs3a, hs1,2, hs3b and hs4-containing region reduces *Myc* upregulation

**Figure 4:**
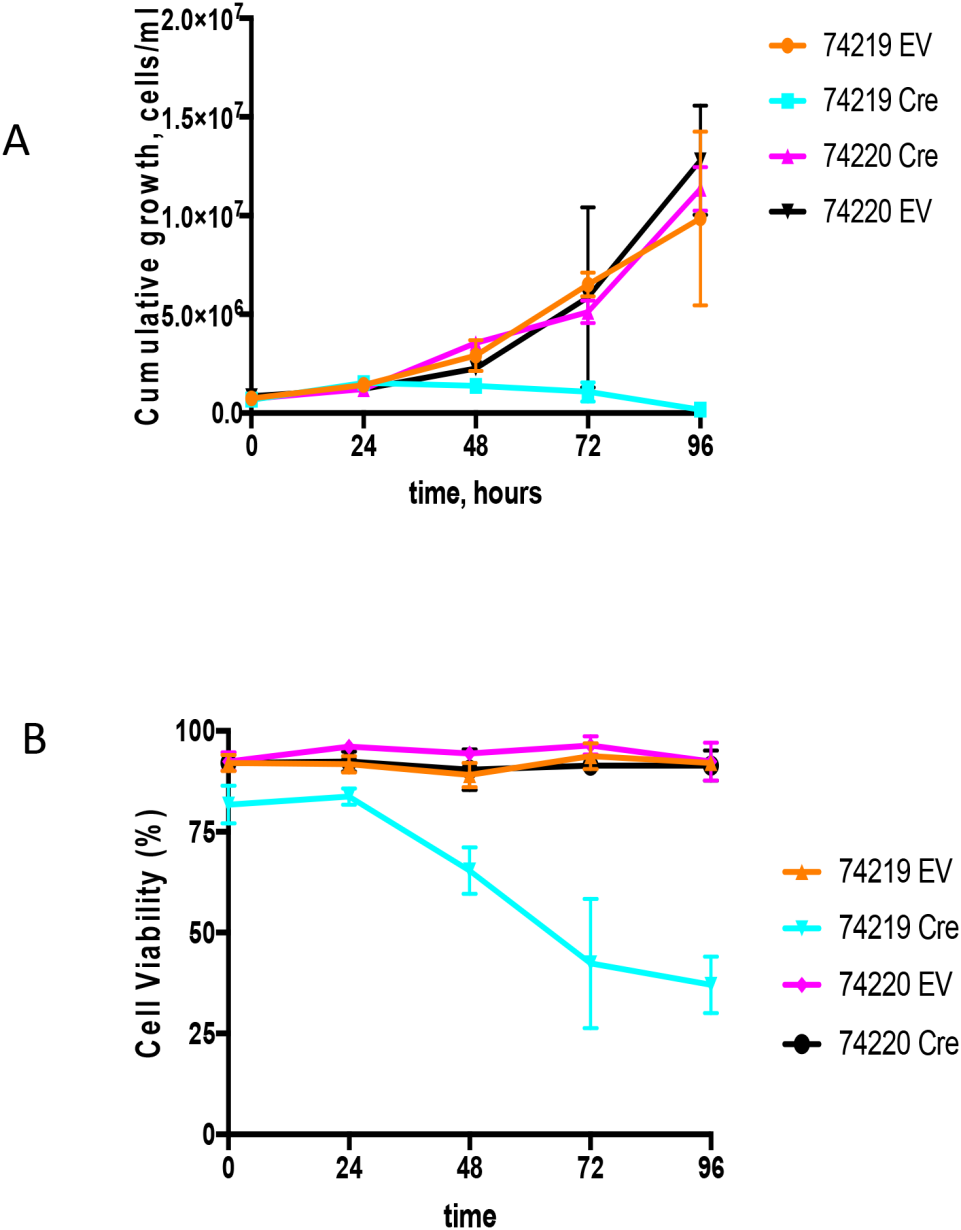
Growth analyses of PCT cells *in vitro* upon Cre-mediated deletion of the *Igh3’RR* **(A)** Shown are the cumulative growth curves after normalization for dilution at each subculture. PCT 74219 has a translocation between transgenic *Igh* and *Myc*, and PCT 74220 has translocation between endogenous *Igh* and *Myc*. Transgenic *Igh* has loxP sites flanking *Igh3’RR* enhancers. PCT cell lines were infected with an empty vector (EV) or Cre-expressing (Cre) retroviruses. 4-OHT was added in the culture medium and growth was monitored by cell counting for 96 h. **(B)** Cell viability curve. Time course effect of Cre-mediated *Igh3’RR* deletion on cell viability was determined by counting cells using trypan blue staining. All values are presented as mean± SD.

Cell lines bearing *Myc* translocations with either the ARS*/Igh*11 transgene (schematics of the molecular structure of the transgene and *Myc*/*Igh-TG* translocations are presented on Supplementary Figure 6) or endogenous *Igh* [28] were transduced with Cre-ER-expressing or empty retroviral vectors (EV) [29]. 4-OHT treatment triggers nuclear translocation of Cre-ER and recombination of loxP sites in target DNA resulting in complete deletion of hs3a, hs1,2, hs3b and hs4 enhancers. There was no effect of 4-OHT treatment on either the EV controls or PCT 74220, the cell line with the endogenous *Igh/Myc* translocation T(12;15) (Figure 4). We analyzed two cell lines with *Igh* TG-containing T(7;15) translocation (74219 and 74160). Both underwent growth arrest starting at 48h. Due to insufficient number of cells for PCT 74160 we present detailed growth curve and viability data for PCT 74219 only (Figure 4A). Cells stopped proliferating 24 hours post treatment and their viability was also reduced such that by 96 hours it was 37±7% (Figure 4B). Cre-mediated deletion was detectable starting as early as at 12 hours and was mostly complete by 48 hours (Supplementary Figure 7A). For the 74219 cell line, *Myc* expression was assayed using semi-quantitative RT-PCR and normalized for β-actin. At 72 hours, *Myc* transcripts were significantly decreased (Supplementary Figure 7B). Expression of GAPDH, a Myc target gene, was also decreased. For another PCT cell line with T(7;15) 74160, we performed qPCR and *Myc* expression decline was similar (Supplementary Figure 7C).

## DISCUSSION

Activity of various regulatory elements embedded within the *Igh* locus is modulated through differentiation stage-dependent recruitment of various transcription and chromatin remodeling factors and exerted through long-range interactions achieved by chromatin looping [4, 6, 38, 39, 40, 41, 42]. There is longstanding interest in dissecting the role of individual enhancers in various recombinational, mutational and epigenetic perturbations affecting the *Igh* locus at different stages of B-cell differentiation. So far, it has been shown that at the mature B-cell stage, targeting of AID, initiation of switch region transcription and recombination as well as subsequent expression of immunoglobulins are heavily, but not entirely, dependent on *Igh*3’*RR* activity (reviewed in [6]).

The discovery that individual enhancer units embedded within *Igh*3’*RR* synergistically interacted with each other and were sequentially activated was instrumental for understanding the relationship between its structure and function [43, 22]. hs4 (termed by Garot et al [22] as the *Igh3’RR* distal module) is necessary to maintain BCR expression for maturation from the bone marrow to the mature MZ and FO splenic B-cells. Upon transition to more mature B-cell stages and engagement in antigen recognition, the germinal center reaction and subsequent plasma cell differentiation, control over most of the *Igh* locus regulatory mechanisms, SHM, CSR, and antibody production, gradually shifts to the hs1,2 enhancer and its palindromic 3a and 3b modules (or proximal module) [1]. From these studies, it can be concluded that hs1,2 is most active in plasma cells.

Several studies uncovered significant differences in both transcription factor recruitment and active chromatin landscapes in the *Igh* locus upon induction of plasma cell differentiation of B-cells or in malignant plasma cells. For example, Pax5 plays a critical role in the regulation of *Igh*3’*RR* activity [44]. As detected using Chip-seq, Pax5 binds the hs1,2 and hs4 elements as well as CTCF sites at the superanchor region [40, 45]. In mature murine B-cells the most strongly enriched Pax5 peak is at hs4 [45]. Importantly, in B-cells Pax5 inhibits hs1,2 activity through suppression of PU.1 binding [44, 46]. This inhibitory action is lifted at the plasma cell transition when Pax5 expression is abolished.

With the identification of hs1,2,3,4 as an *Igh* locus control region, it was proposed that it may be also responsible for *Myc* deregulation in Burkitt lymphomas and mouse PCT [47]. In the majority of these tumors, the *Myc* gene is fused to switch regions of the *Igh* locus downstream of *Eμ*, therefore making the *Igh*3’*RR* the main candidate to drive *Myc* overexpression. Activity of the individual enhancer units and their combinations was tested in various cell lines using reporter constructs driven by *Myc* or λ1 promoters. It was shown that hs1,2 is most active in PCT and the highest activity of hs4 was recorded in pre-B cells [47, 48]. Interestingly, hs4 activity varied between PCT cell lines and was highest in S194 and much weaker in MPC11, TEPC1165 and J558. Nevertheless, hs1,2 or hs4 in combination with the hs3 palindrome, or the more complete hs1,2,3,4 combination, reproducibly enhanced transcription of reporter constructs to the highest levels. On the other hand, when the hs1,2,3a deletion mutant was analyzed, it was shown that hs3a and hs1,2 are not essential for high levels of *Igh* expression or for spontaneous class switching in a PCT cell line [49]. Analysis of rare PCTs with inversions of the *Igh* locus on the translocated allele (ABPC 26, ABPC 60, 26485 and 33433) or insertion of 3’*Igh* into the *Myc* locus (BPC4) showed that integrity of the whole *Igh*3’*RR* was always retained [Kovalchuk, unpublished], [50]. It can be concluded that the most efficient *Igh* regulation can be achieved only by concerted action of all four enhancer elements. However, hs1,2 and hs4 may compensate for each other, especially at the late stages of B-cell differentiation.

The role of the *Igh*3’*RR* in *Myc* deregulation and lymphomagenesis was extensively studied in many mouse knock-in and transgenic models (reviewed in [20]). However, only Gostissa et al [21] in their study assessed effects of partial deletion of *Igh3’RR* on the occurrence of *Igh*/*Myc* translocations and their progression from pre-oncogenic lesions towards lymphomas. They showed that in the absence of hs3b-4 at the pro-B cell stage, *Eμ* can effectively drive expression of *Myc* in the context of non-reciprocal 12;15. Peripheral B-cell lymphomas deficient for Ligase 4 and XRCC4 on the WT *Igh*3’*RR* background carry T(12;15) translocations that fuse *Myc* to Sμ (removing *Eμ*). However, hs3b-4-deficient mice developed T-cell lymphomas only and no *Igh*/*Myc*-positive B-cell lymphomas. This was despite the fact that hs3b-4-deleted alleles were efficiently targeted by AID, had abundant DSB in Sμ and even were fused with *Myc* in precursor cells. This finding is consistent with the fact that at the B-cell differentiation stage to which peripheral lymphomas corresponded, activity of only the hs1,2 and 3a enhancers was not sufficient to drive deregulated *Myc* expression in *Igh*/*Myc* translocation-positive precursors [21]. It is also important to note that in the model used by Gostissa et al [21], lymphomas originate from transitional B-cells, precluding analysis of the role of *Igh*3’*RR* in post-GC lymphomas or PCT.

Taking in consideration the sequential activation of enhancer elements and the stronger role of hs1,2 at the plasma cell stage, we thus decided to determine if Gostissa et al [21] findings could be revised in the mouse PCT model. Consistent with the reduced number of DNA breaks overall, and specifically those targeting downstream switch regions due to class switch defects in hs3b-4 KO mice [21], T(12;15)-positive tumor precursor cells in these mice may have accumulated in lesser numbers and preferentially carried breakpoints in *Sμ.* However, in the mouse PCT model lacking hs3b-4 enhancers, these tumor precursors were able to progress to the terminal stage of B-cell differentiation when hs3a and 1,2 enhancers alone became fully capable of deregulating *Myc*. This resulted in the outgrowth of fully malignant PCTs with no change in latency but with a lower incidence compared to WT and Het controls.

When we compared WT and hs3b-4 KO allele utilization for T(12;15) in the hs3b-4^+/-^ PCTs, no bias was detected. The patterns of breaks in the KO allele were similar to those in hs3b-4^-/-^. This contrasts with the lymphoma model [21] where arising tumors always targeted the WT allele for the chromosomal translocation. In addition to the lowered tumor incidence, in both hs3b-4^+/-^ and hs3b-4^-/-^ PCTs, the pattern of breaks in the *Igh* locus differed from that observed in *Igh3’RR*-proficient mice. This likely follows the altered CSR-accessibility pattern in non-malignant B-cells carrying a *Igh3’RR* defect, with decreased targeting of *Igh* downstream *C* genes and *S* regions.

Interestingly, the three of nine hs3b-4^+/-^ tumors that utilized the hs3b-4 KO allele for the T(12;15) lost the untranslocated WT *Igh* allele and duplicated the translocated chromosome. Only one of the WT-targeted group similarly duplicated the T(12;15) but retained the mutant untranslocated Chr 12. This may suggest that *Myc* expression levels are not high enough for a precursor cell to become fully transformed. However, we did not observe T(12;15) chromosome duplications in the hs3b-4^-/-^ PCTs. It is plausible that high *Myc* expression levels in hs3b-4^+/-^ can be achieved by T(12;15) duplications and in the hs3b-4^-/-^ by some other mutation and/or rearrangement that we did not detect.

It is still not clear how precursor B-cells differentiate into malignant plasma cells. There could be several pathways with some of them requiring higher *Myc* expression at the B-cell stage where it is achieved by the above-mentioned duplication. Also, the biology and maturation of B-cells that express the WT versus the KO allele must be as different from each other as the hs3b-4^-/-^ mouse is from the hs3b-4^+/-^.

Detailed analysis of *Myc* expression levels, P1/P2 promoter shift and immunoglobulin secretion did not reveal any significant differences between hs3b-4^-/-^ and WT PCT. This likely confirms that, similarly to the situation in non-malignant B-cells, the hs3b and hs4 enhancers have a major role at the GC stage, but their absence can be later compensated in differentiated plasma cells, then supporting full *Igh* expression or *Myc* deregulation in PCTs.

Regulatory roles of the very proximal *Igh* DNAse hypersensitivity sites occupied by CTCF (superanchor) are yet to be fully defined. In contrast, the four major *Igh3’RR* enhancers hs3a, hs1,2, hs3b and hs4 were proposed to play the critical role in *Myc* deregulation upon chromosomal translocations. We decided to determine if deletion of only these four major enhancers would result in abolishing *Myc* deregulation in the context of *Igh*/*Myc* translocation. For that, we utilized PCT cell lines that were developed from tumors that arose in the ARS/*Igh11*-transgenic mouse model [28]. The cell lines carry *Igh/Myc* translocations involving either the transgenic *Igh* (inserted into Chr 7) where the *Igh3’RR* is flanked with *loxP* sites or the endogenous Chr 12 WT allele. Cre-mediated deletion of the *Igh*3’*RR* caused loss of elevated *Myc* expression, downregulation of the *Myc* target gene *Gapdh*, block of proliferation and decreased viability.

Upon plasma cell transition, hs3a and hs1,2 enhancers acquire the leading role in regulating the activity of the *Igh* locus. Our experiments prove that in plasma cell tumors hs3a and hs1,2 are fully capable of governing *Myc* deregulation and P1/P2 promoter shift in the context of T(12;15) as well as Ig secretion from the non-translocated chromosome. However, deletion of all four major *Igh*3’*RR* enhancers - hs3a, hs1,2, hs3b and hs4 - in PCTs is detrimental to expression of a cis-linked oncogene and demonstrates that such translocated oncogenes are really *Igh*3’*RR*-driven.

## MATERIALS AND METHODS

### Mice

Frozen embryos of Δ*hs3b4* (hs3b-4^-/-^) mice on 129 genetic background [23] were obtained from Centre National de la Recherche Scientifique, France and rederived at the Cryopreservation & Assisted Reproduction Laboratory, NCI-Frederick. The mice were bred and maintained in a barrier-protected conventional colony under NCI IACUC approved protocol, LG024. Genotyping for the defective allele was carried out by PCR using the following primers:

HS3bHS4 delta Forward: 5' TGTCCCCCATTT CTTGTCAT 3'

HS3bHS4 delta Reverse: 5' GACCCTGTCCT ATGGCTGAC 3'.

HS4 5’: 5' CCAAAAATGGCCAGGCCTAGG 3'

hs3b-4^-/-^ stock mice were crossed with BALB/c-Bcl-xL transgenic mice [24]. The F1 hybrids were then mated to raise F2 offspring and from this population Bcl-xL-hs3b-4^-/-^, Bcl-xL-hs3b-4^+/-^ and Bcl-xL-hs3b-4^+/+^ mice were selected for use in induction studies. For tumor transplants we used 2-3-month old athymic nude BALB/c mice (BALB/c-nu/nu) that were purchased from Charles River (NCI-Frederick, MD).

### Plasmacytoma induction

Plasmacytomas (PCT) were induced by a single i.p. injection of 0.4 ml pristane (2,6,10,14–tetramethylpentadecane; Aldrich, Milwaukee, WI, USA) into 2-3-month-old mice. The mice were monitored, beginning between days 30-40 after injection and every two weeks thereafter, for the appearance of PCT cells in the peritoneal exudate as previously described [12]. Oil granuloma tissue from tumor-bearing mice was injected in pristane-primed BALB/c-nu/nu recipients. Cell lines were established from primary tumors or first-generation transplants by culturing in complete RPMI 1640 medium with 10 ng/ml of in-house produced recombinant mouse IL-6. Tissues were fixed in Fekete's modified Telleysniczky's fluid, embedded in paraffin, sectioned at 4–5 μm and stained with hematoxylin and eosin by Histoserv, Inc. (Gaithersburg, MD). Histology images were viewed with an Olympus BX41 microscope (10× to 100× objectives) and photographed with an Olympus DP71 camera (both from Olympus, Waltham, MA). DP controller software version 3.3.1.292 was used for image acquisition. Histopathological diagnoses were made using established criteria [25]. Briefly, cells constituting a mature plasmacytic PCT are medium-sized sIg− cIg+ CD138+ atypical plasma cells with pyroninophilic cytoplasm, round eccentric nucleus with marginated chromatin and one or several nucleoli. Tumors exhibiting less mature, plasmacytoid or plasmablastic features contain mixture of medium to large-sized plasma cells and plasmablasts with less cytoplasm, a more central nucleus, and more prominent nucleoli than plasmacytic PCT.

### Cytogenetic analysis

FISH analysis on metaphase chromosomes was performed as previously described [12]. BACs [*Igh* (189A22), Cμ (18D13), *Myc* (D15Mit17)] were labeled with either biotin or digoxygenin using corresponding nick translation kits (Roche, Indianapolis, IN). The hs3b-4 *Igh3’RR* WT probe was prepared as previously described [21] and labeled with biotin using the nick translation kit. Probes were visualized with streptavidin Alexa Fluor 568 (Molecular Probes, Invitrogen, Carlsbad, CA) or sheep anti-digoxigenin-fluorescein Fab fragments (Roche, Indianapolis, IN). Images were acquired on an Olympus IX81 fluorescent microscope and processed using Slidebook software v.5.0.25 (Intelligent Imaging Innovations, Denver, CO).

SKY was performed using a SKYPaint probe kit from Applied Spectral Imaging (Carlsbad, CA) according to the manufacturer’s instructions. SKY image acquisition was performed by using the SpectraCube SD300 (Applied Spectral Imaging, Carlsbad, CA) connected to an epifluorescence microscope (Axioskop 2, Carl Zeiss, Germany). Image acquisition and processing were achieved using HiSKY software (v4.0, Applied Spectral Imaging, Carlsbad, CA).

### Amplification of translocation breakpoints, VDJ rearrangements and switch region junctions

Ascitic cells and samples of oil granulomas were harvested at necropsy. Total DNA extraction was performed using the Puregene DNA isolation kit (Gentra Systems, Inc., Minneapolis, MN). For detection of translocation breakpoints and switch region junctions, one round PCR amplifications was performed as described [14] using Expand High Fidelity PCR system (Roche, Indianapolis, IN). V_H_ gene amplification was performed as previously described [26]. PCR products were extracted from 1.5% agarose gels using QIAquick Gel extraction kit (Qiagen, Chatsworth, CA) and sequenced either directly or after cloning using TOPO TA kits (Invitrogen, Carlsbad, CA) at the NCI CCR Genomics core facility using BigDye Terminator Version 1.0 cycle sequencing kit from ABI (Carlsbad, CA). Sequence alignments were performed using IgBLAST (http://www.ncbi.nlm.nih.gov/igblast) or MacVector software v15.03 (MacVector, Inc., Apex, NC).

### RNA isolation, quantitative RT-PCR analysis

Cells were resuspended in TRI reagent (Sigma-Aldrich, St. Louis, MO) and total RNA was extracted using the RNeasy Mini kit (74104; Qiagen, Chatsworth, CA). cDNA was synthesized from 2 μg of total RNA by SuperScript III First-Strand Synthesis kit using oligo(dT)_20_ primers (Invitrogen, Carlsbad, CA). 7900HT Sequence Detection system (Applied Biosystems), SYBR Green PCR Master Mix (Applied Biosystems, Foster City, CA) and gene-specific primers were used for amplification and real-time PCR analysis of cDNA samples (5 ng) in triplicates, and results are presented relative to those of β actin.

Primers used for qPCR:

mmu-MYC P1F: 5’-CTGCGCTGCTCTCAGCTG-3’

mmu-MYC P2F: 5’-GACTCGCTGTAGTAAT 00TCCAG-3’

mmu-MYC Exon2R: 5’-TCATAGTTCCTGTT GGTGAAGTT-3’

mmu-MYC Exon3F: 5’-GGCAGGGTCCTGAAGC AGAT-3’

mmu-MYC Exon3R: 5’-CCGCCTCTTGTCGTT TTCCT-3’

mβactin reverse: 5’-CAGCCTGGATGGCTACG TACAT-3’

mβactin forward: 5’-CCGTGAAAAGATGACC CAGATC-3’

Primers used for RT-PCR:

Myc5'Exon3: 5’-CGGCGTAGTTGTGCTGGTGAGT-3’

Myc3'Exon2: 5’-CCGCGCCCAGTGAGGATATCT-3’

GAPDH-rev: 5’-CACATTGGGGGTAGGAACACGGAAGG-3’

GAPDH-fw: 5’-CGGTGCTGAGTATGTCGTGGAGT-3’

mβactin reverse: 5’-CAGCCTGGATGGCTACGTA CAT-3’

mβactin forward: 5’-CCGTGAAAAGATGACCCA GATC-3’

### Paraprotein analysis

Serum paraproteins were detected in 10 μl of undiluted serum using Hydragel 15 HR electrophoresis kit on a Hydrasys apparatus (Sebia, France) according to the manufacturer’s instructions. Monoclonal bands were detected visually on stained gels.

Paraprotein isotypes in sera or ascitic fluids were determined by ELISA using isotype-specific goat anti-mouse IgM, IgG (IgG1, IgG2a, IgG2b, IgG3), and IgA labeled with horseradish peroxidase (Southern Biotech Associates, Birmingham, AL). Immulon 2 HB plates (Dynex Technologies, Chanitlly, VA) were coated with serum or ascitic fluid at dilutions of 10^−3^ to 1.28 × 10^−5^. Plates were read on a microplate reader SpectraMax Plus (Molecular Dynamics, Sunnyvale, CA) at 450 nm and data analyzed using v5.3 SoftmaxPro software of the same company.

### RNA-Seq

One microgram of RNA was used as input for the TruSeq Directional Total RNA-Seq Sample Preparation Kit (Illumina, Inc, San Diego, CA) utilizing Ribo-Zero H/M/R to deplete ribosomal RNA. Final library size distribution was assessed on BioAnalyzer DNA 1000 chips (Agilent Technologies, Santa Clara, CA). Libraries were quantified using the Kapa SYBR FAST Universal qPCR kit for Illumina sequencing (Kapa Biosystems, Boston, MA) on the CFX96 Real-Time PCR Detection System (Bio-Rad Laboratories, Inc, Hercules, CA). The libraries were diluted to 2 nM stocks and pooled equitably for sequencing.

The 2 nM pool of libraries was prepared for the first round of sequencing by denaturing and diluting to an 11 pM stock for clustering to the flow cell. On-board cluster generation and paired-end sequencing was completed on the HiSeq 2500 (Ilumina, Inc, San Diego, CA) using a TruSeq Rapid PE Cluster Kit and TruSeq Rapid SBS Kits (Illumina, Inc, San Diego, CA) for 2 x 101 bp sequencing.

The paired end sequence reads were prepped by first removing any adapter sequences with CutAdapt v1.12, then low quality sequences were filtered and trimmed using the FASTX Toolkit. Remaining reads were then mapped to the *M. musculus* genome mm10 using HiSat2 v2.0.5 with strict pairing required. Differential expression analysis was performed using DESeq2 with low/no expressing genes removed and the standard median ratio normalization method applied. Chimeric transcripts were determined using Tophat-fusion v2.1.1 (5). A likely candidate list was generated by filtering out those with less than 100 supporting reads spanning the chimera. In addition, we generated a modified reference mm10 genome containing M-MLV insert in order to visualize the retroviral integration.

Reads are deposited in the SRA database under project ID PRJNA481385.

### Retroviral transduction and 4-OHT treatment

PCT cell lines used for retroviral infection were developed from primary tumors arising in *Igh* BAC transgenic line 820. This line carries a single copy of the ARS*/Igh*11 transgene with LoxP sites inserted 5’ of hs3a and 3’of hs4 enhancers [27]. The construct is integrated on Chr 7 and competes with endogenous *Igh* locus for being translocated with *Myc* in PCTs induced by pristane injection on *Bcl-xL*-transgenic background [28].

Cre-ERT2-Puro and empty vector retroviral constructs [29] were provided by Marcus Müschen, UCSF School of Medicine. Retroviral packaging and infection were carried out according to a standard protocol. Stably-transduced clones were grown in complete RPMI medium containing 15 μg/ml of puromycin (Sigma). Cells were washed in fresh medium with the addition of 5 μg/ml of 4-OHT (Sigma), seeded at 2 x10^5^ per well into a Costar 12-well plate (Corning Inc, Corning NY) and incubated at 37°C for 96 hours. Every 24 hours, half of the culture volume was substituted with fresh medium containing all the above-mentioned ingredients. Cells removed from the culture were enumerated on a Cellometer Auto T4 (Nexcelom Bioscience, Lawrence MA) and their viability was assessed using trypan blue dye exclusion. Then the cells were pelleted and their RNA and DNA were extracted for subsequent analyses. Cre-mediated deletion was detected by disappearance of a *loxP* site in transgene-specific hs3a using PCR followed by DraI digestion according to [27].

## SUPPLEMENTARY MATERIALS FIGURES AND TABLES












