# Supplementary material for: 3’Igh enhancers hs3b/hs4 are dispensable for Myc deregulation in mouse plasmacytomas with T(12;15) translocations
Source: Oncotarget. 2018 Oct 2;9(77):34528–42. doi: 10.18632/oncotarget.26160 (PMC6195379; doi:10.18632/oncotarget.26160)
Supplement: Supplementary file 1 [file oncotarget-09-34528-s001.pdf]

3'*Igh* enhancers hs3b/hs4 are dispensable for *Myc* deregulation in mouse plasmacytomas with T(12;15) translocations

SUPPLEMENTARY MATERIALS

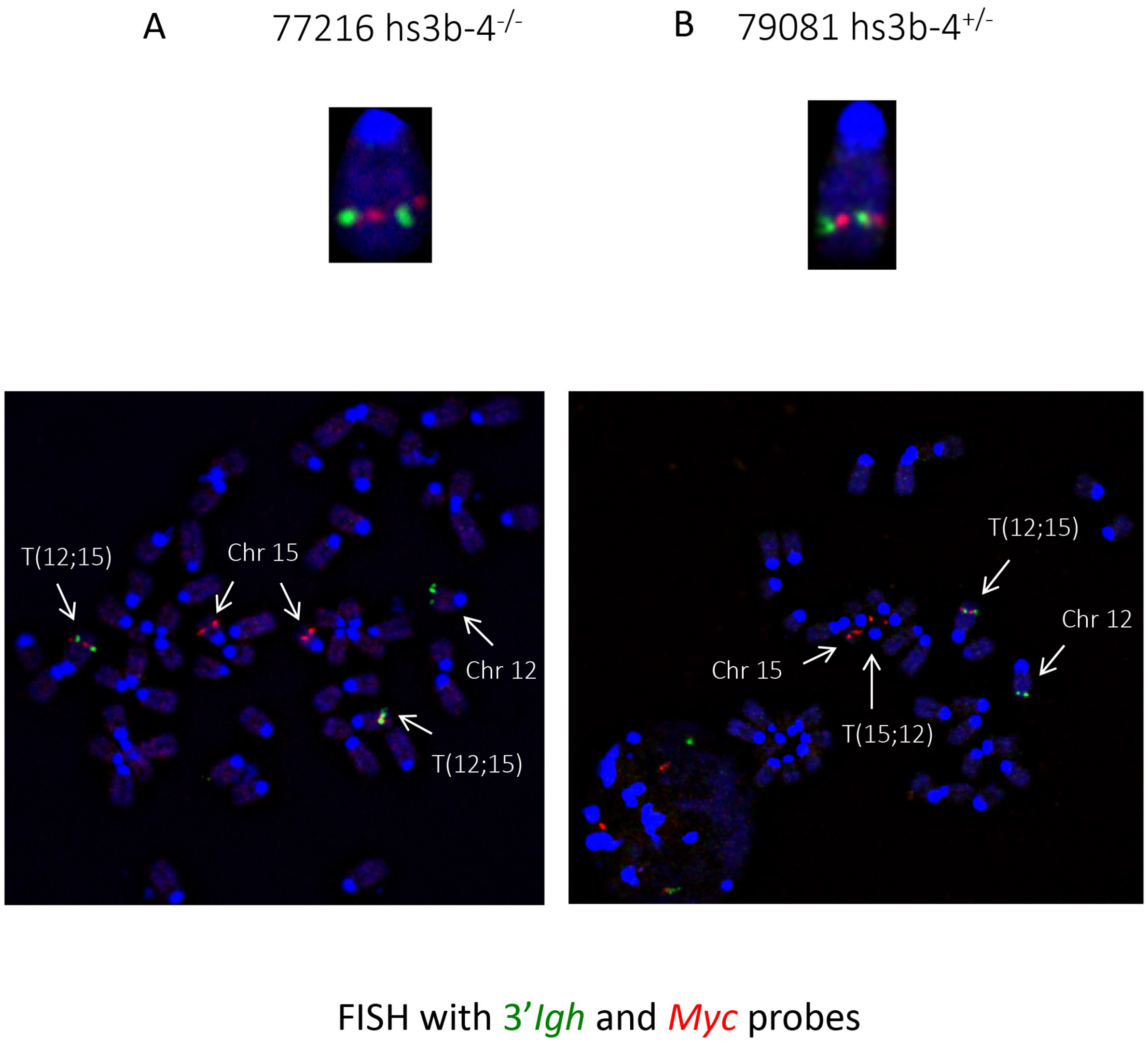

Supplementary Figure 1: Plasmacytomas from hs3b-4$^{-/-}$ and hs3b-4$^{+/-}$ harbor reciprocal *Igh/Myc* translocations T(12;15). Representative FISH with *Igh* (green) and *Myc* (red) probes showing presence of T(12;15) in metaphases of hs3b-4$^{-/-}$ (panel **A**) and hs3b-4$^{+/-}$ (panel **B**) PCT cell lines. Magnified clippings of T(12;15) chromosomes are shown above each corresponding metaphase. *Igh* probe detects both WT and hs3b-4 KO alleles. It hybridizes to normal and translocated T(12;15) chromosome 12. *Myc* probe splits and detects normal chromosome 15, and two reciprocal products T(12;15) and T15;12).

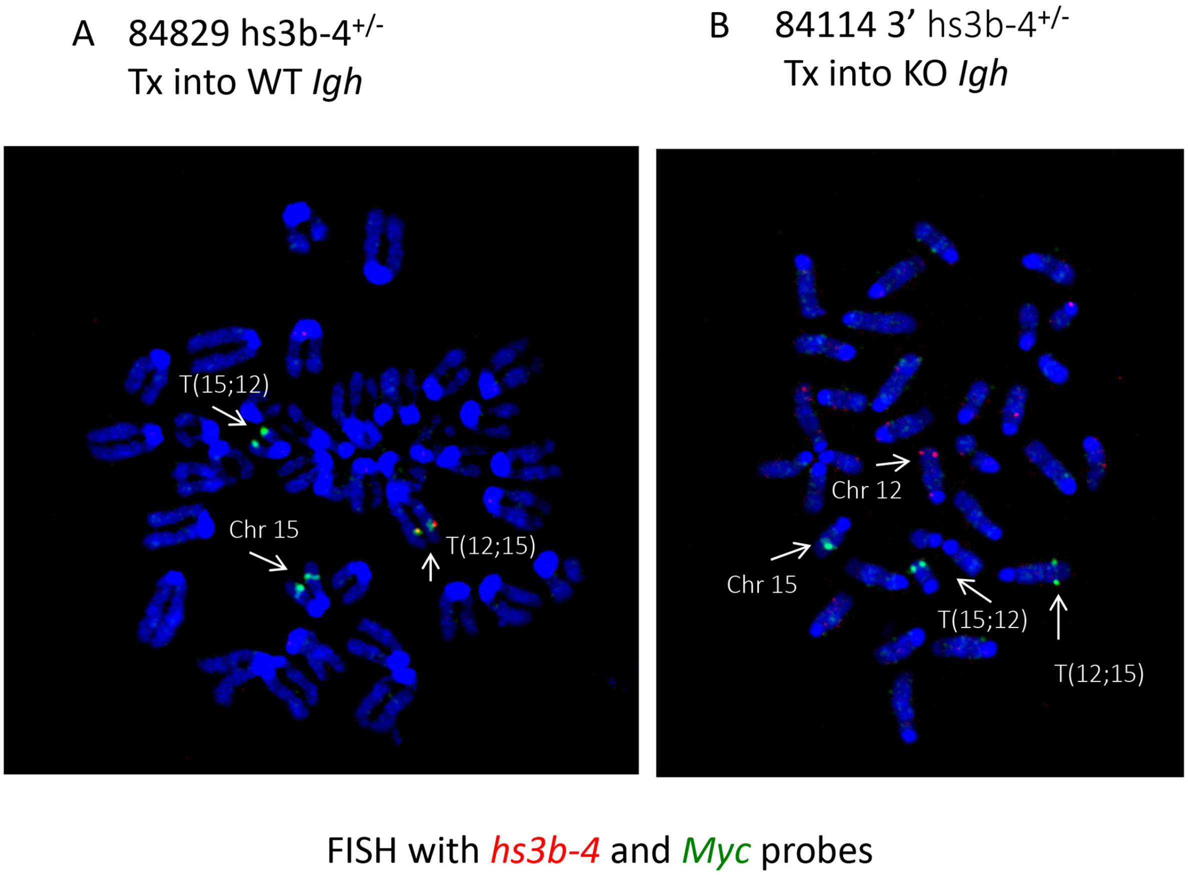

Supplementary Figure 2: In hs3b-4$^{+/-}$ both *Igh* alleles are targeted with T(12;15). Representative FISH with hs3b-4 (red) and *Myc* (green) probes. hs3b-4 probe detects only WT *Igh* allele and does not hybridize to the hs3b-4 KO allele. *Myc* probe splits and hybridizes to normal and translocated chromosomes 15. In PCT 84829 (Panel **A**) there is co-localization of both probes on WT Chr12;15. In this case, the hs3b-4 KO allele on Chr 12 is not detected by either of the two probes. In PCT 84114 (Panel **B**) hs3b-4 probe hybridizes only to WT normal Chr 12 and not to T(12;15) where only the *Myc* probe is visible.

A 82794 hs3b-4$^{+/-}$ Tx in KO

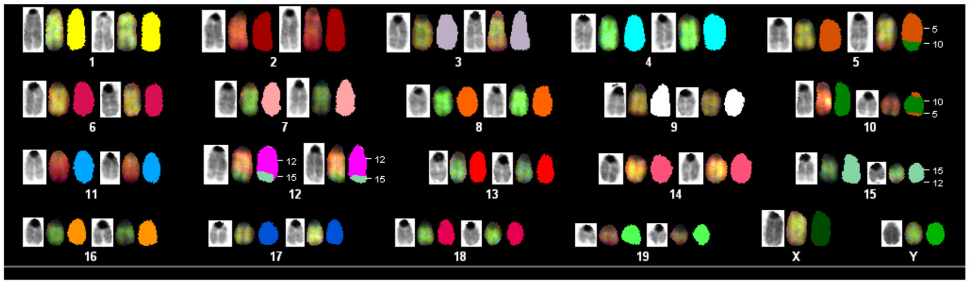

40, XY, T(5;10), T(10;5), -12, +T(12;15), T(15;12)

B 77216 hs3b-4$^{-/-}$

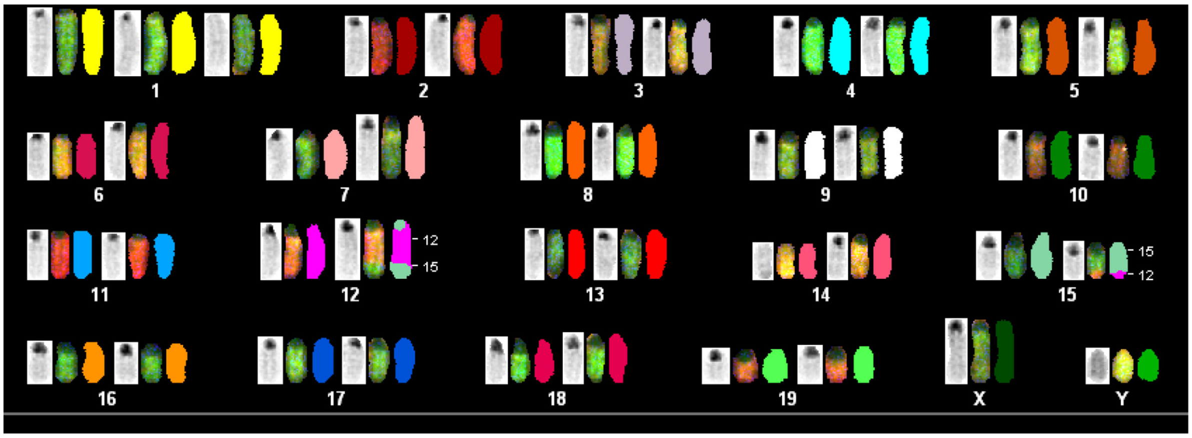

41, XY, Del(6), T(12;15), T(15;12)

Supplementary Figure 3: SKY analysis of hs3b-4$^{+/-}$ and hs3b-4$^{-/-}$ PCT cell lines. **(A)** Karyotype of hs3b-4$^{+/-}$ cell line 82794 analyzed by SKY shows a reciprocal T(12;15) translocation involving chromosomes 12 and 15. In this particular case, hs3b-4 KO Chr 12 was targeted by translocation and duplicated, while WT Chr 12 was lost (proven by FISH and not presented on this figure). The presence of only single copies of both normal Chr 15 and T(15;12) likely precludes the possibility of two independent T(12;15) translocations. Also present is a reciprocal T(5;10) translocation. **(B)** A representative karyotype of hs3b-4$^{-/-}$ PCT cell line. PCT 77216 has a nearly diploid karyotype with T(12;15), T(15;12) and trisomy for Chr 1.

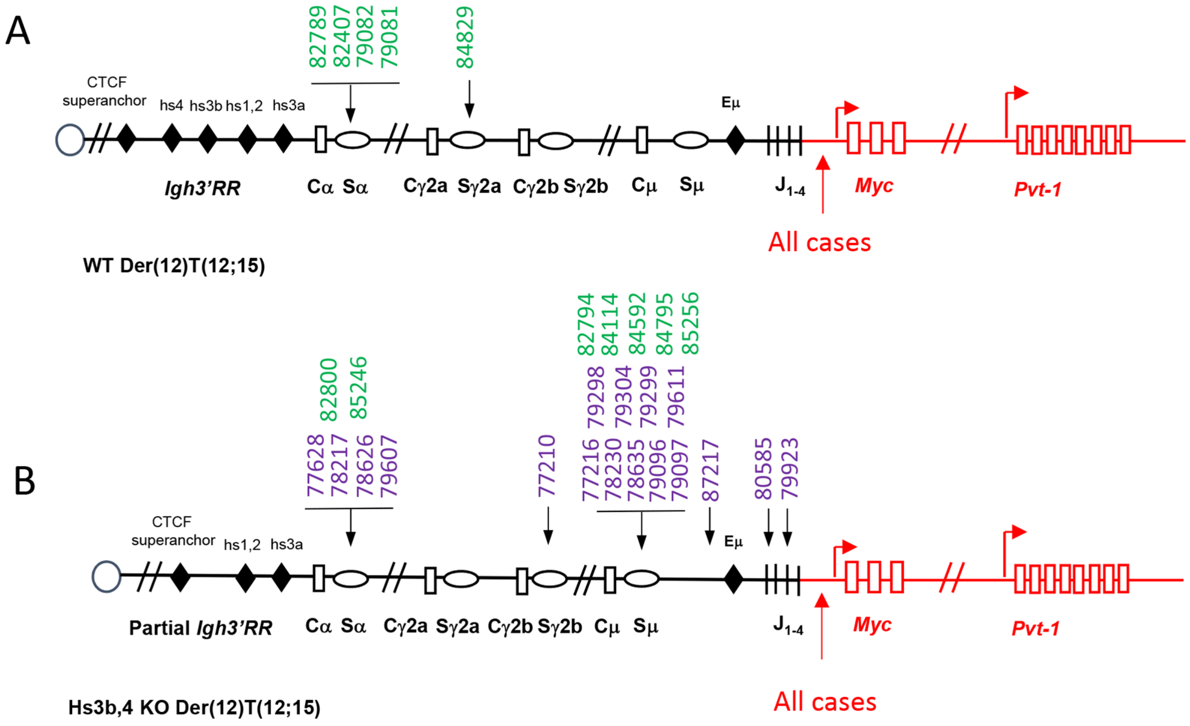

Supplementary Figure 4: Breakpoint map. Schematic representation of *Igh* (depicted in Black) and *Myc/Pvt1* (depicted in Red) loci. Location of breakpoints is indicated by arrows pointing down (in *Igh*) and up (in *Myc*). Individual cases are labeled in purple for the hs3b-4$^{-/-}$ and in green for the hs3b-4$^{+/-}$ groups. In all cases, breakpoints in *Myc* were localized in the immediate 5'*Myc* promoter upstream region. **(A)** Breakpoint locations for hs3b-4$^{+/-}$ PCTs with T(12;15) targeting the WT *Igh* allele. **(B)** Breakpoint locations for hs3b-4$^{+/-}$ and PCTs hs3b-4$^{-/-}$ with T(12;15) targeting the KO *Igh* allele.

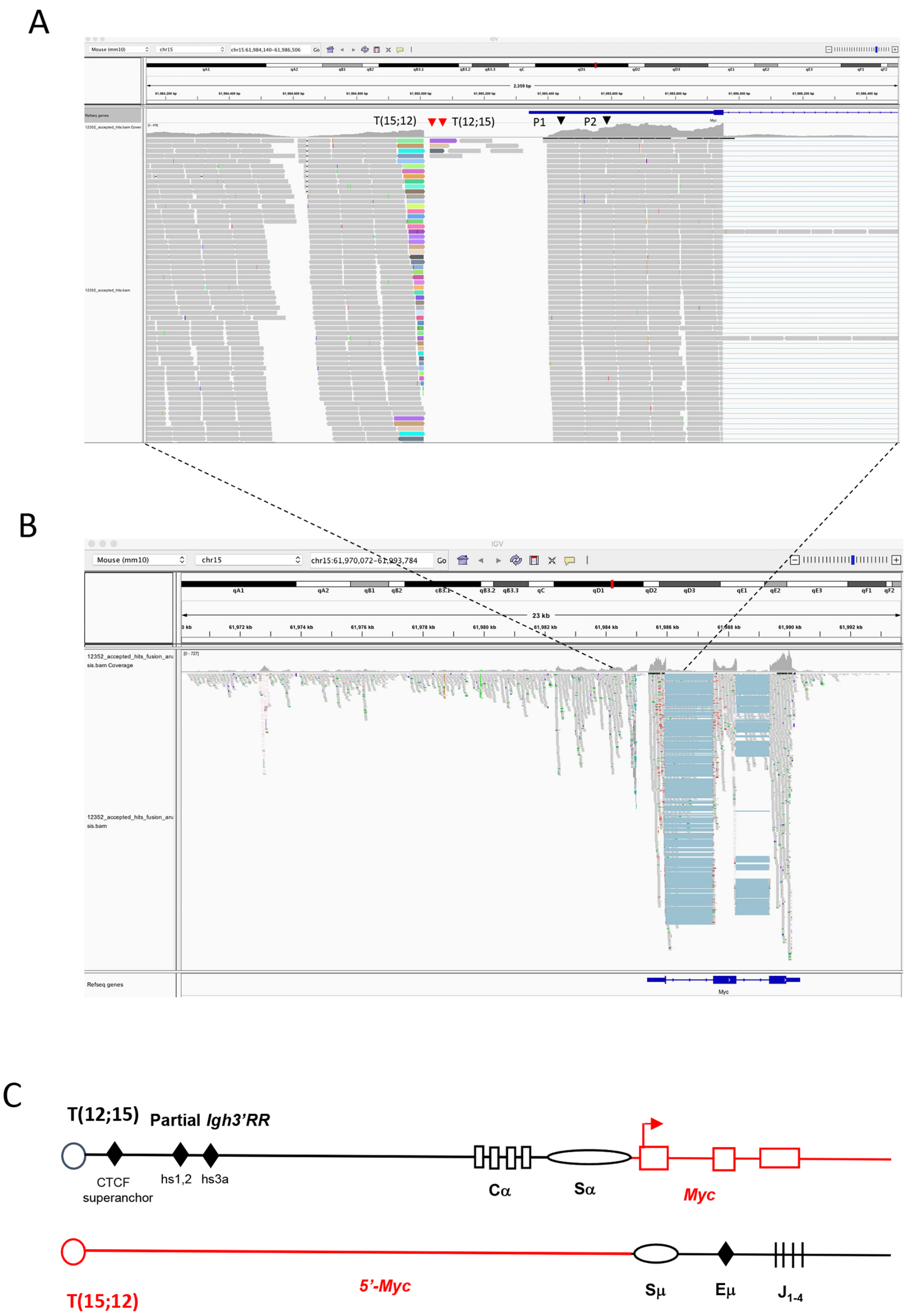

Supplementary Figure 5: Integrative genomics viewer (IGV) visualization of alignments of RNA-seq reads to mm10 reference genome. (A) Exon1 of *Myc* with flanking sequences. Two major *Myc* promoters P1 and P2 located in 5'UTR are marked with black arrows pointing down. Location of T(12:15) and reciprocal T(15;12) breakpoints in immediate 5'*Myc* promoter upstream region of hs3b-4$^{-/-}$ PCT 77628 is marked by red arrows pointing down. Colored reads show chimeric transcript junctions between *Myc* and *Igh*. **(B)** Zoomed out view of the *Myc* locus. Most of the reads within the *Myc* gene body originate from P1 promoter. Transcriptional activity in far 5' *Myc* is driven by the *Eμ* enhancer located on the reciprocal T(15;12) chromosome. **(C)** Schematic representation of *Igh/Myc* chimeric loci formed after a reciprocal T(12;15) translocation in PCT 77628.

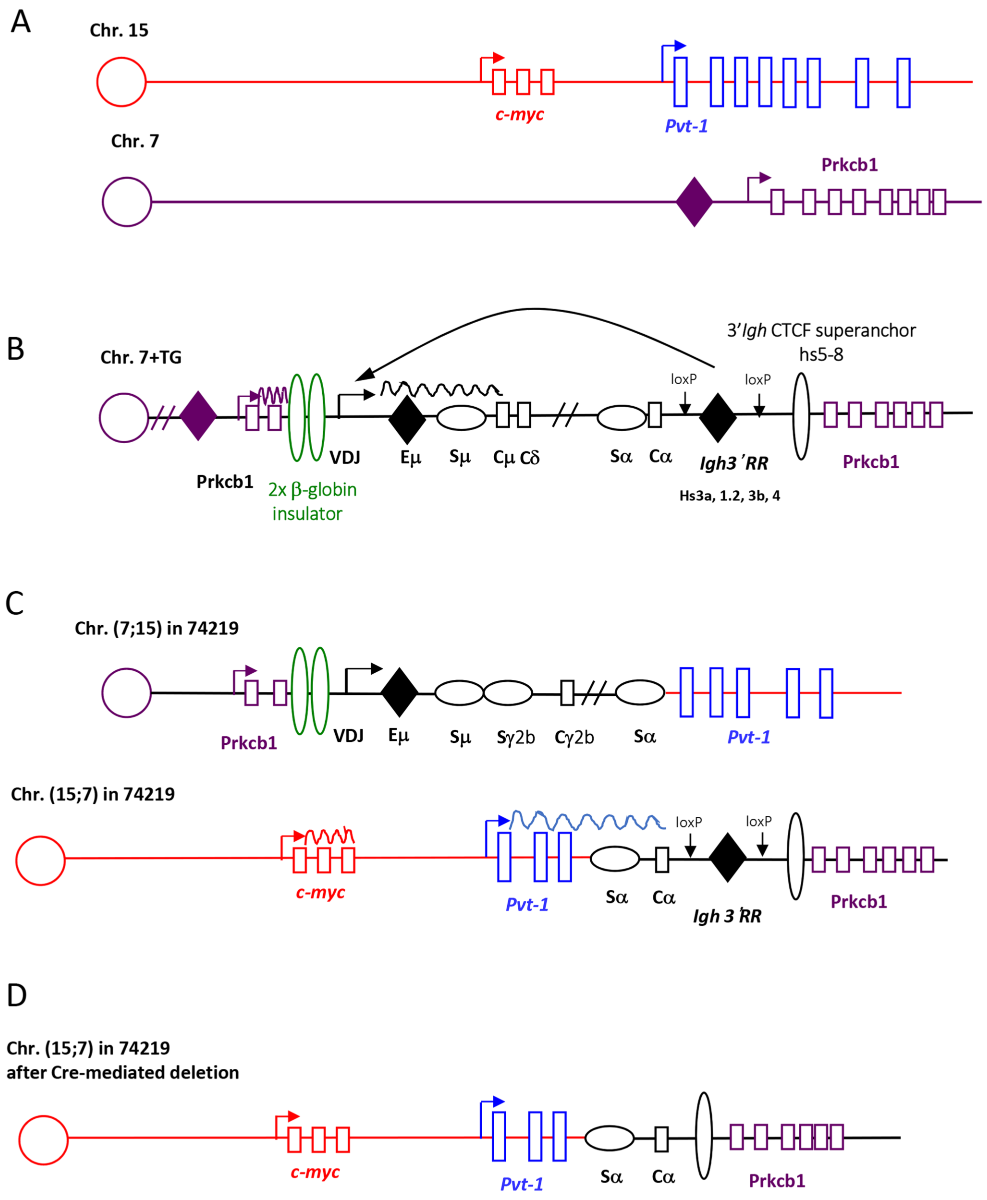

Supplementary Figure 6: Schematic representation of the structure of *Igh*/*Myc* translocation in a PCT cell line derived from ARS/*Igh11*-transgenic mice. **(A)** Structure of normal mouse chromosomes 15 and 7 showing *Myc*/*Pvt*-1 and *Prkcb1* loci. **(B)** Single copy of ARS/*Igh*11 BAC transgene integrated into *Prkcb1* locus on chromosome 7 in a mouse line 820. **(C)** Reciprocal T(7;15) translocation involving the transgene and intron 3 of *Pvt-1* in PCT 74219. **(D)** Cre-mediated deletion of *Igh*3'*RR*.

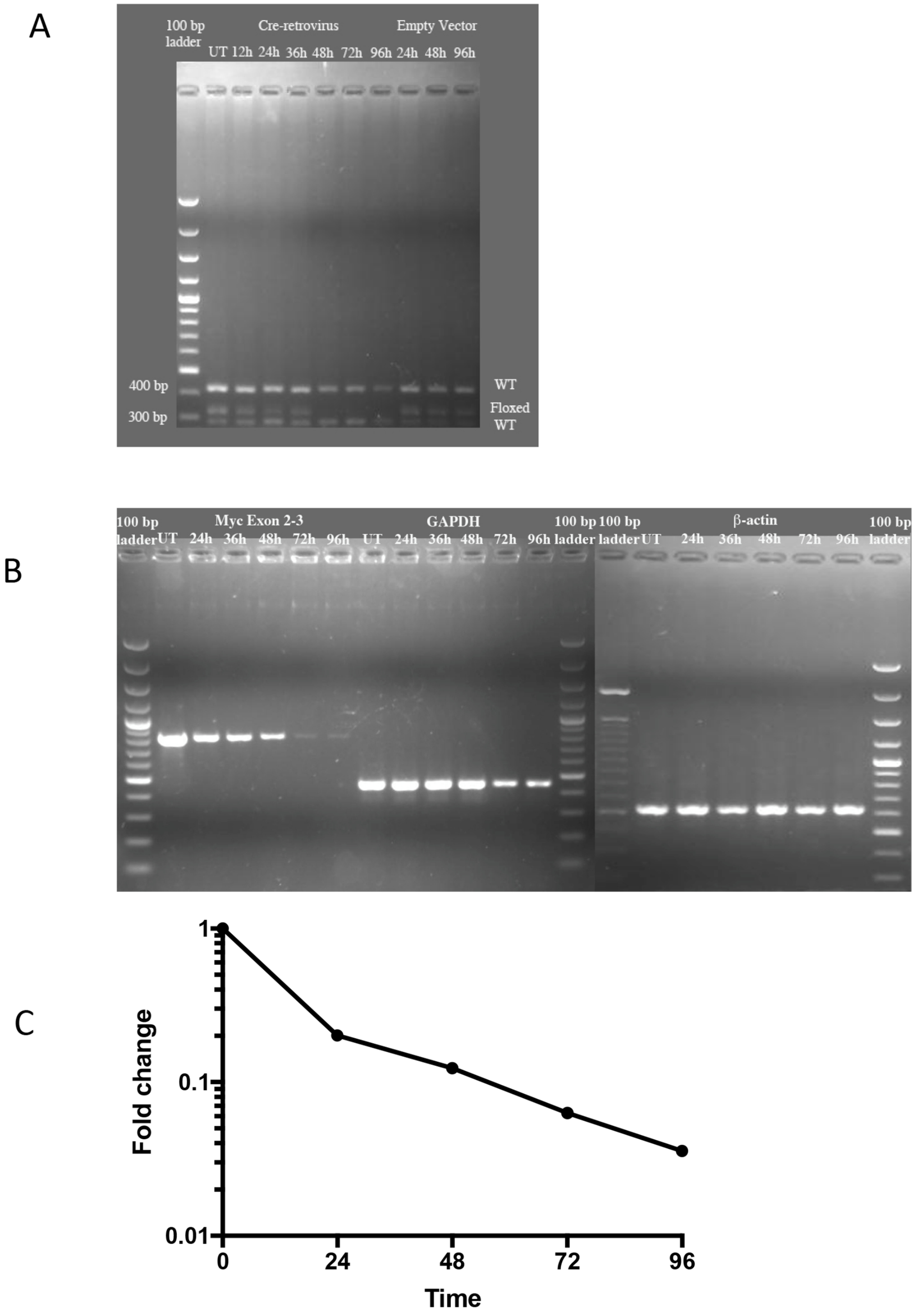

Supplementary Figure 7: Loss of translocated *Myc* gene overexpression upon Cre-mediated deletion of cis-linked hs3a, 1.2, 3b, 4 enhancers. **(A)** DraI digestion of PCR products fractionated on an agarose gel showing disappearance of the floxed band. In untreated samples, digestion of PCR products with DraI generates three bands: 421, 274 and 312 bp. The first two correspond to the 129 WT allele that has an internal DraI site. The 312 bp transgenic band includes the loxP site. This band disappears with 4-OHT treatment as loxP sites recombine and original sequences are deleted. **(B)** Relative quantification of *Myc* and its target gene *Gapdh* (left), and *β*-actin (right) expression using semi-quantitative RT-PCR before, 24, 36, 48,72 and 96 hours after 4-OHT treatment in PCT 74219. **(C)** qPCR analysis of *Myc* expression normalized to β-actin in T(7;15)-positive PCT 74160 before, 24, 48,72 and 96 hours after 4-OHT treatment.

Supplementary Table 1: Summary of findings in hs3b-4-/- and hs3b-4+/- PCT cell lines

See Supplementary File 1

Supplementary Table 2: Characterization of T(12;15) breakpoint junctions in hs3b-4-/- and hs3b-4+/- PCTs

See Supplementary File 2

Supplementary Table 3: Characterization of VDJ junctions in hs3b-4-/- and hs3b-4+/- PCTs

See Supplementary File 3

Supplementary Table 4: Characterization of switch region junctions in hs3b-4-/- PCTs

See Supplementary File 4

Supplementary Table 5: Characterization of immunoglobulin secretion by hs3b-4-/-, hs3b-4+/- and hs3b-4+/+ PCTs

See Supplementary File 5
